# Effects of a brief pre-admission telephone reminder on no-show and dropout rates in substance use disorder treatment: a quasi-experimental study

**DOI:** 10.1186/s13011-022-00489-9

**Published:** 2022-08-23

**Authors:** Lisbeth Jensen Gallefoss, Karin Berle Gabrielsen, Siri Håvås Haugland, Thomas Clausen, John-Kåre Vederhus

**Affiliations:** 1 Department of Health and Nursing Sciences, University of Agder, Mailbox 422, 4604 Kristiansand, Norway; 2 Addiction Unit, Sørlandet Hospital Trust, Kristiansand, Norway; 3 Department of Psychosocial Health, University of Agder, Grimstad, Norway; 4 Norwegian Centre for Addiction Research (SERAF), Institute of Clinical Medicine, University of Oslo, Oslo, Norway

**Keywords:** Substance use disorders, Outpatient treatment, Reminder systems, Telephone reminder, Norway

## Abstract

**Background:**

Appointment no-show and early dropout from treatment represent major challenges in outpatient substance use disorder treatment, adversely affecting clinical outcomes and health care productivity. In this quasi-experimental study, we examined how a brief reminder intervention for new patients before their first appointment affected treatment participation and retention. No-shows (not attending any sessions) and dropouts (discontinuation of initiated treatment because of three consecutively missed appointments) were compared between a period with pre-admission telephone calls (intervention) and a period without such reminders (non-intervention).

**Methods:**

Participants were all eligible patients (*N* = 262) admitted to a Norwegian specialist clinic for substance use disorder treatment. We used the Chi-square test for the no-show analysis. Of the eligible patients, 147 were included in a subsequent dropout analysis. We used the number of visits up to 10 appointments as a measure for time to event. Group differences were analyzed using a Kaplan–Meier plot and the log-rank test. To control for relevant sociodemographic variables, as well as substance use and mental distress severity, we used Cox regression.

**Results:**

No-show rates did not differ between the two periods (12% for non-intervention vs. 14% for intervention; χ^2^ = 0.20, *p* = 0.653). Of those consenting to participate in the dropout analysis (*n* = 147), 28 (19%) discontinued treatment within the time frame of 10 appointments, with no differences between the two periods (log-rank test = 0.328, *p* = 0.567). Controlling for baseline characteristics did not alter this finding. In fact, of the registered covariates at baseline, only higher education level was associated with attrition, linked to a reduced risk for dropout (hazard ratio = 0.85, 95% CI = 0.74–0.98, *p* = 0.025).

**Conclusion:**

These findings do not provide support for the systematic use of a brief pre-admission telephone reminder in the current treatment setting.

**Trial registration:**

The study was retrospectively registered 13 Jan 2021 at ClinicalTrials.gov, NCT04707599.

## Background

Substance use disorder (SUD) is a serious clinical condition that can lead to major health problems and affect a wide range of life domains, including professional career, family life, and social relations [1–3]. The societal costs are high, and in Norway as elsewhere, excessive use of alcohol and illegal drugs is an important risk factor for severe illness and premature death [1, 4].

Motivating individuals to initiate and stay in treatment is a priority because treatment completion is strongly correlated with favorable outcomes [5], including lower risk of relapse, improved physical and mental health, and higher employment levels [6]. A report on Norwegian interdisciplinary treatment for SUDs, i.e., specialized treatment for SUDs, cited a 35% increase in outpatient consultations over a 4-year period (2013–2017), and outpatient treatment is now regarded as the first option in most addiction treatment clinics [7, 8]. This structural shift accentuates the challenge of no-shows in successfully initiating treatment. A high prevalence of missed appointments results in underuse of resources in terms of equipment and personnel, increased waiting times for appointments, increased service costs, and poorer treatment outcomes [9]. A recent review found an average missed appointment rate in Europe of 19% across different medical specialties [10]. Missed appointment rates in SUD treatment settings are typically higher than in general health care settings [11]. Obtaining a clear picture of the problem is difficult, however, as authors use different definitions and typically refer to any missed appointment as a “no-show,” regardless of whether the patient presents for later appointments [10]. A straightforward definition of no-show is when a patient never presents at the treatment facility at all.

Even when the patient has initiated treatment, attrition can be high, particularly in outpatient SUD treatment, with dropout rates typically reported at 20%-50% [5, 12–15]. Dropout can be defined as an unplanned discontinuation of an initiated treatment, often because of several consecutively missed appointments [16]. Factors associated with dropout include mental distress [17], main substance of use [18], illicit drug use severity [19], and education level [17, 20]. A few studies have examined dropout in the inpatient setting in Norway [21, 22], but we are not aware of any studies examining dropout among outpatients in Norway. Despite this scarcity of data from outpatient settings, the Norwegian Directorate of Health has expressed concerns about high dropout rates [23], and some government-funded incentives have been launched, to examine dropout prevalence and address the problem more systematically [7, 24]. The early treatment stages appear to be a particularly vulnerable time [13, 25], with a substantial portion of dropouts occurring within days to a few weeks of initiating therapy [11, 15].

Various methods to reduce no-show and missed appointment rates have been researched and clinically implemented. One such method is the use of reminder systems, described as a simple, adaptable, and cost-effective intervention to increase the likelihood that patients will attend their appointments [25]. One review of reminder systems within general health care services demonstrated a benefit of all types of reminders, including standard text messages and personal phone calls (“reminder plus”), in reducing non-attendance at health appointments, irrespective of patient group and targeted health condition. The review indicated that the “reminder plus” was sometimes more effective than the simple reminder in reducing non-attendance. Patients cited receiving a reminder before the appointment as a sign of positive and genuine concern from the health care provider [9]*.* Nevertheless, how reminders before the first appointment affect attendance remains uncertain and has not been extensively explored in research for the SUD population [25].

In our addiction unit, we initiated use of an extended reminder routine entailing a telephone call to the patient before the scheduled first appointment (i.e., “reminder plus”). The primary aim of this study was to test our hypothesis that this routine would reduce no-show rates. A secondary aim was to examine whether this intervention also would affect dropout rates after treatment initiation.

## Methods

### Setting

Interdisciplinary treatment for SUD in Norway is financed publicly and largely organized within the specialist health care services (in the hospitals), and outpatient services are provided at the cost of a deductible for the patient. This study was part of a national quality improvement initiative by the Norwegian Health Authority to reduce dropout from SUD treatment and was conducted in 2015 at the addiction treatment unit at Sørlandet Hospital, Norway. At this unit, a simple reminder in the form of an SMS text message is standard beginning with the second appointment, following patient consent and phone number verification. No standard procedure for giving reminders before the first appointment had been established before this study.

### Design and intervention

We used a quasi-experimental (off–on) design to compare a period without a telephone reminder to a period with a telephone reminder [26]. Each inclusion period lasted for 10 weeks. During the first phase (April to June), standard hospital procedures were followed, with only a written appointment letter to all new referred patients. During the second phase (August to October), therapists attempted to call all patients 2–3 days before the first appointment. The phone call was limited to a short, informative exchange in which the therapists introduced themselves and reminded patients of their upcoming appointment. The therapists were asked to make up to 2–3 attempts to contact the patient.

### Participants and procedures

**Fig. 1:**
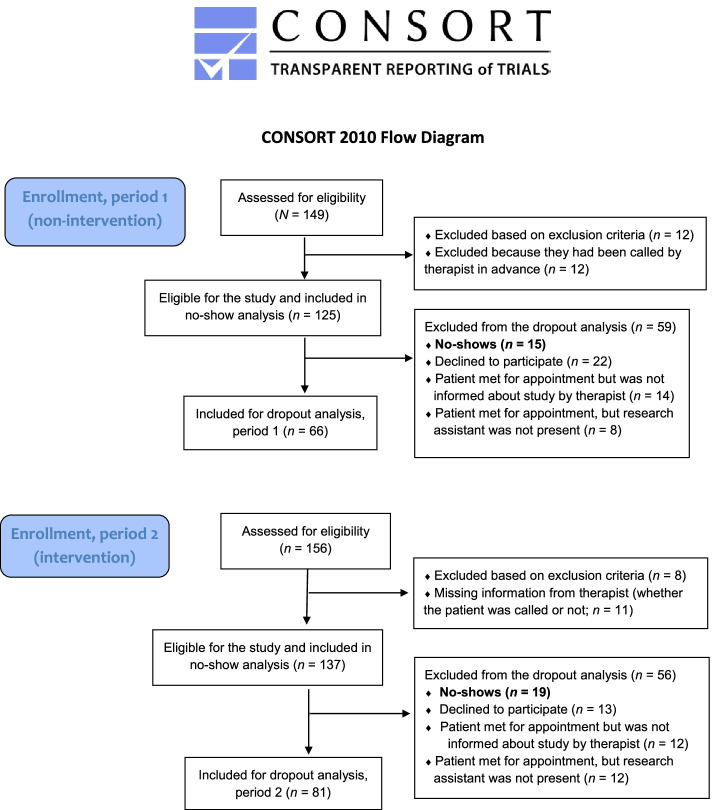
Flow chart of study participants (*N* = 262)

Participants were recruited among new referrals to outpatient clinics at the hospital. We obtained ethical permission to base the no-show analysis on all patients who were eligible for the study, i.e., whether or not a later informed consent was obtained (see below). The involved therapist noted on a form whether they had phoned and reached the patient. Patients enrolled in the clinic’s opiate maintenance programs and patients with gambling disorders were excluded from participation. For the subsequent dropout analysis, patients who kept their first appointment were informed about the project during their first session and invited to participate. To prevent expectation bias, the patients received general information about the study, focusing on quality improvement of the treatment given at the clinic. Thus, patients were blinded to the specific aim of examining the effect of the pre-admission phone call, which was implemented as a standard procedure in the second period of the study, the reminder phone call phase. Informed consent was obtained from those who wished to participate. In addition to the above-mentioned exclusion criteria, we did not include patients who showed signs of being under the influence of alcohol or drugs at intake and had difficulty responding to questionnaires because of, for example, language issues. A flow chart (Fig. 1) shows the number of patients included in the two analyses.

### Measures

#### Primary outcome

The primary outcome was no-show rates during the “off” period compared with the “on” period. “No-shows” were defined as patients who never showed up for treatment.

#### Secondary outcome

The secondary outcome was treatment dropout rate during the “off” period compared with the “on” period. We used the number of visits up to 10 appointments as a time frame for counting dropout. A typical frequency in our unit is one appointment a week, meaning the time span for counting dropout was an average of 10 weeks for the 10 appointments, with some individual adjustment of appointment frequency in agreement with the patient. Patients received new appointment invitations even after two missed appointments, but a third missed appointment led to a discharge. We used a strict definition of “dropout,” defining it as discontinued treatment, i.e., the patient had initiated treatment but was discharged because of three consecutively missed appointments, whether they had phoned to reschedule or not. Thus, dropout was defined as three missed appointments within a time frame of 10 sessions. Administrative patient records were used to confirm attendance at the first appointment and whether the patient subsequently completed treatment or attended scheduled appointments within the 10-session time frame.

#### Additional variables used in the dropout analysis

For the dropout analysis, we collected demographic data such as age, sex, and diagnosis. In addition to controlling the findings for personal characteristics, i.e., basic socio-demographics, it was important to examine whether the association between the intervention phases and the dependent variable (drop-out) remained after taking into account relevant covariates. Severity of substance use and level of mental distress, i.e., mental health severity, may be factors in treatment dropout [16]. We assessed perceived severity of substance use with a subscale from the Survey of Readiness for Alcoholics Anonymous Participation (SYRAAP) [27, 28] and measured mental distress with the Hopkins Symptom Checklist 10 (SCL-10).

The five questions of the SYRAAP scale, e.g., “Using substances has interfered with my ability to deal with everyday problems,” were rated on a five-point Likert-type response format, from strongly disagree to strongly agree. A mean score was computed, with a higher score indicating greater severity. A patient sample at a Norwegian detoxification clinic had an average of 4.2 on this scale [28]. Thus, a score ≥ 4 on the SYRAAP scale was viewed as reflecting a serious substance use problem.

The SCL-10 consists of 10 items mapping present levels of anxiety (4 items) and depression (6 items). Responses were scored on a four-point scale ranging from 1 to 4. The global severity index constitutes the average of items, with the highest score indicating the highest distress. The clinical cut-off was set at 1.85. The scale has been shown to be a valid indicator of mental distress and validated in a Norwegian setting [29, 30].

The data collection for the dropout analysis took an average of 10–15 min per patient, and each patient was reimbursed with a gift certificate of NOK 150 (USD 15).

### Statistical analyses

Descriptive statistics were used to characterize participants. Because of the quasi-experimental design, we used Student’s t-tests and the Chi-square test to examine differences in baseline characteristics between the two periods (non-intervention and intervention) at baseline. The Chi-square test also was used to analyze the no-show rate. To compare dropout rates between the intervention and non-intervention conditions, we used a Kaplan–Meier plot and the log-rank test. We used Cox regression analysis as a secondary analysis to control for covariates, such as sociodemographics and substance use severity. We first undertook preliminary univariate analyses of the sociodemographic variables. For multivariable regression, we added variables that were significant in the univariate analyses, and in addition to controlling the analysis for sociodemographic factors, we entered SUD diagnosis, disorder severity and mental distress as relevant covariates [16]. The multivariable regression model applied a simultaneous entry of variables (the ‘‘enter’’ method). Results are reported as hazard ratios (HRs) with 95% confidence intervals (CIs). All statistical analyses were performed using IBM SPSS, version 26 [31].

## Results

### Primary outcome

The treatment participation/no-show analysis was based on all eligible patients (*n* = 262) during the full study time frame (Fig. 1). In the intervention period, therapists called 125 of 137 patients (91%) but made contact with only half of them (67 patients), with a mean call duration of 4.7 min. Of the 262 eligible patients, 12% in the non-intervention period (15 of 125) and 14% in the intervention period (19 of 137) were no-shows (χ^2^ = 0.20, *p* = 0.653).

### Secondary outcome

**Fig. 2:**
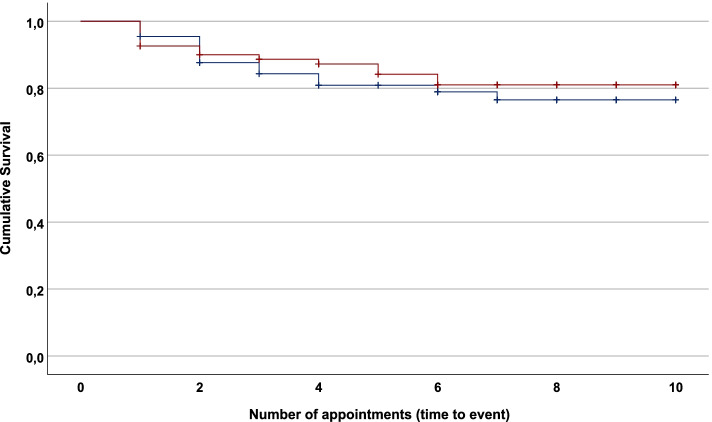
Kaplan–Meier plot showing dropout rate for the non-intervention period (blue line, without telephone reminder; *n* = 66) and intervention period (red line, with telephone reminder; *n* = 81). Log-rank test = 0.328 (*p* = 0.567)

The sample included in the dropout analysis (*n* = 147) had a mean age of 36 years, and 24% (35 of 147) were women (Table 1). Most (57%) were diagnosed with a SUD related to illicit drugs, and 43% had an alcohol use disorder. In total, 28 of 147 patients (19%) discontinued treatment within the time frame of 10 appointments. There were 14 dropouts during each period (21% non-intervention and 17% intervention, χ^2^ = 0.364, *p* = 0.546). The Kaplan–Meier plot with separate dropout rates for each period is shown in Fig. 2 (log-rank test = 0.328, *p* = 0.567).

**Table 1:** Characteristics of study participants in the dropout subsample (*N* = 147), with data presented as *n* (%) or mean (± standard deviation)

Characteristics	Non-intervention *n* = 66	Intervention *n* = 81	*p* ^a^
Age, years	36 (± 14)	36 (± 11)	0.844
Sex, female	16 (24)	19 (24)	0.911
Education, years	11.1 (± 2.8)	12.0 (± 2.4)	0.035
Living alone or unstable living conditions	38 (58)	48 (59)	0.837
Welfare benefits or no income	51 (77)	59 (73)	0.720
Substance use disorder diagnosis			
Alcohol use disorder	25 (38)	38 (47)	0.271
Drug use disorder	41 (62)	43 (53)	
Substance use severity ^b^	3.8 (± 1.0)	3.7 (± 0.9)	0.633
Mental distress ^c^	2.18 (± 0.78)	2.09 (± 0.70)	0.436

^a^ *P* values from Student’s t-test for continuous variables and the Chi-square test for binomial variables

^b^ SYRAAP severity sub-scale, range 1–5

^c^ Hopkins Symptom Checklist 10, range 1–4; clinical cut-off, 1.85

### Sociodemographic factors and covariates

The mental distress mean score was above the clinical cut-off, with more than half of the participants (79 of 147) reporting mental distress levels above this cut-off at baseline. The patients’ baseline data did not differ significantly between the two periods (Table 1), except that the intervention group had completed one more year of education, on average (12 vs. 11 years; Table 1).

In the multivariable Cox regression analysis controlled for clinical characteristics registered at baseline, the two periods still did not differ (Table 2). Substance use and mental health severity did not contribute to explaining dropout. The only covariate that significantly contributed to explaining dropout was education level, with a higher level associated with reduced risk for dropping out (HR = 0.85; 95% CI = 0.74–0.98, *p* = 0.025). This result did not change when the analysis was controlled for age and sex (not shown).

**Table 2:** Cox regression analysis examining predictors of dropout (*N* = 147)

	Univariate analyses		Multivariable analysis	
Factors	HR (95% CI)^a^	*p*	HR (95% CI)^a^	*p*
Condition (non-intervention as reference)	0.81 (0.39–1.70)	0.575	1.11 (0.51–2.41)	0.787
Age	0.99 (0.96–1.02)	0.451	–	–
Sex	0.75 (0.33–1.71)	0.496	–	–
Living alone or unstable living conditions	1.27 (0.59–2.76)	0.539	–	–
Welfare benefits	1.44 (0.65–3.19)	0.366	–	–
Education	0.84 (0.74–0.96)	0.009	0.85 (0.74–0.98)	0.025
Substance use severity^b^	1.32 (0.85–2.07)	0.220	1.33 (0.83–2.12)	0.239
Substance use diagnosis (drug use disorder)	2.37 (1.01–5.58)	0.048	2.13 (0.88–5.11)	0.092
Mental distress^c^	0.93 (0.56–1.55)	0.789	0.85 (0.49–1.47)	0.560

^a^ Cox regression; hazard ratios (HRs) with 95% confidence intervals (CIs)

^b^ SYRAAP severity subscale, range 1–5

^c^ Symptom Checklist, global score index

## Discussion

Regarding our primary outcome, the practice of calling new patients in addition to a standard admission procedure did not lead to lower no-show rates, which we had hypothesized it would. The intervention also did not influence the secondary outcome, i.e., it did not reduce early dropout in this outpatient setting. Substance use severity and mental distress were not associated with dropout, but higher education was associated with reduced dropout rate.

Our reminder condition (phone call) did not seem to boost attendance rates or affect attrition. These findings are in line with those of several other studies from general health care services that have reported similarly neutral results [32, 33] but contradict others showing a benefit of pre-treatment phone calls on no-show rates and later dropout [11, 34, 35]. Relatively little research has been conducted on this topic in the area of specialized substance use treatment [11, 25], and patients with SUDs belong to a particularly disadvantaged and vulnerable population that may benefit from more extensive reminder routines [9, 34, 36]. Donohue et al. [36] found a 29% higher attendance at the intake session after a 15-min informational phone call versus a few minutes of brief orientation. In their study, the intensive intervention group also received a follow-up call within 7 days after the first session to discuss possible concerns. These findings correspond to later research indicating that how the intervention is implemented may be important. According to Lefforge et al. [37], the calls should be made within 48 h of the initial call to the clinic and should entail certain elements, such as an explanation of the upcoming program and expected treatment, a discussion of obstacles to attendance, and a review of patient expectations. It was also recommended that the contacts conclude with a verbal commitment from the patient to attend the session [37]. In the present real-world setting, we chose the brief reminder condition in an effort to optimize the cost–benefit ratio and lessen the burden on the therapists.

In this study, we found dropout rates of 19%, in line with previously observed lower end estimates [5, 12–15]. In publicly financed SUD treatment systems, patients know that they will eventually be offered at least one extra appointment regardless of prior attendance or non-attendance. However, recipient welfare benefits in Norway are often contingent on active participation in treatment, and patients are required to pay a fee similar to the deductible if they fail to notify the clinic that they cannot attend the session. This requirement may serve as an economic incentive to remain in treatment and to notify the unit in advance and may help explain why the original dropout rates were at the lower end of the estimates, as noted above. It is also of note that total attrition figures should include both no-shows and dropout. The combined attrition of no-shows at the first appointment and subsequent dropout in this study was 24% within the first 10 sessions, with all eligible patients as the denominator.

The association between the main independent variable (non-intervention and intervention periods) and the dependent variable (dropout) remained after accounting for substance use and mental health severity, neither of which was independently related to dropout. Mental health severity has been reported as a predictor for dropout in a previous Norwegian study [17], but that work focused on inpatients. Outpatient treatment may involve patients with less mental distress, which could explain why mental distress was unrelated to dropout in the present study despite a mean mental distress score above the clinical cut-off. A high severity of substance use has been reported to increase intrinsic motivation to seek help [38], and severity of substance use typically does not explain dropout from SUD treatment [13], corroborating the present findings. In agreement with other reports of an association between education level and dropout rates [17, 39–41], we found that lower education level was predictive of dropout. Olfson et al. suggested that patients with higher education may be more responsive to certain forms of psychotherapy [41]. It is conceivable that the organizational skills necessary for attaining a higher education can support adherence to structure and routines in other areas of life, such as keeping appointments and completing therapy. In the present study, the intervention group had a higher level of education than the group that received no telephone call. In line with our findings that higher education predicts higher treatment compliance, it is likely that the dropout rate in the non-intervention group would have been even lower had it not been for this group difference.

Overall, seen in the context of other divergent findings in the areas of dropout/reminder systems, the current results suggest that patients with SUDs are a highly complex and vulnerable patient group who can be difficult to engage in treatment [15]. Ambivalence, fluctuating motivation, and reduced ability to plan ahead may make regular attendance at appointments difficult [42]. The needs of the most vulnerable of these patients may be difficult to meet in a standard outpatient clinic where there may be a waiting period between the referral and the initial appointment [15, 43]. Some of these patients may benefit from a more customized, flexible setup with faster turnaround and better availability [7], such as the Flexible Assertive Community Treatment model (FACT). FACT provides prolonged, extensive, and combined services to persons with severe substance use or mental disorders [44]. Such a low-threshold service is resource demanding but could prove a cost-effective option from a longer-term socioeconomic perspective, at least for some SUD patients. Considering the vulnerable patient group in focus, one could also see this as an ethical imperative, regardless of the uncertainty of its cost-effectiveness [45]. Indeed, FACT has since been implemented at the clinic, partly as a consequence of the current findings, in an effort to organize SUD treatment as a continuum of care woven into the health care system, better adapted to each patient’s individual needs [15].

### Methodological considerations

The results of this study reflect its setting in an outpatient service in a Norwegian hospital and can likely be generalized to similarly organized public health care systems. However, some limitations exist. Only half of the patients could be reached and included in the intervention period, as described. As also noted by Lefforge et al. [37], this proportion may have limited the potential effectiveness of the reminder. However, the real-world conditions of the study and the choice to analyze the data by condition allocation contribute to increased external validity and generalizability of the findings. Although the study was conducted 7 years ago, no structural changes in the organization of SUD treatment have been implemented during this period, and we consider the findings as still valid. Because ours is a non-randomized study, we cannot exclude that confounding variables may have influenced the results, as could have the short time span of only 10 appointments. However, our regression model could, to a very low degree, explain dropout from treatment, and we believe it is unlikely that a longer observational period would have yielded different results. Future studies of dropout in the outpatient setting should use a randomized design with a longer study period to examine potential long-term effects of a reminder intervention.

## Conclusions

The present findings do not support the systematic use of a brief pre-admission telephone reminder in the current treatment setting.

## Data Availability

Anonymized data used for this analysis can be obtained from the last author upon reasonable request.

## Abbreviations

CI: Confidence interval
FACT: Flexible Assertive Community Treatment
HR: Hazard ratio
SCL-10: Hopkins Symptom Checklist 10
SUD: Substance use disorder
SYRAAP: Survey of Readiness for Alcoholics Anonymous Participation
